# Rational Polyligand Design for G‑Quadruplex Multimer Stabilization

**DOI:** 10.1021/acs.jcim.6c01189

**Published:** 2026-08-29

**Authors:** Deniz Mostarac, Flavio Scipione, Mattia Trapella, Luca Bertini, Valeria Libera, Jussara Amato, Bruno Pagano, Lucia Comez, Alessandro Paciaroni, Laura Lupi, Cristiano De Michele

**Affiliations:** † School of Chemistry, University of Edinburgh, EH9 3FJ Edinburgh, U.K.; ‡ Department of Physics, University of Rome La Sapienza, 00185 Rome, Italy; § Department of Physics and Geology, 9309 University of Perugia, 06123 Perugia, Italy; ∥ Department of Pharmacy, University of Naples Federico II, 80131 Naples, Italy; ⊥ CNR—Istituto Officina dei Materiali (IOM), 06123 Perugia, Italy; # Dipartimento di Matematica e Fisica, Università Roma Tre, 00185 Rome, Italy

## Abstract

G-quadruplexes (G4) are noncanonical nucleic acid secondary structures formed by guanine-rich sequences that can form higher-order multimers under appropriate conditions and represent attractive targets for multivalent molecular recognition. Here, we propose a rational ligand design strategy based on multivalency, in which ligand molecules are covalently linked into polymer-like macromolecules. Using coarse-grained molecular dynamics, we show that these “polyligands” intercalate G4 multimers more efficiently than their monomeric counterparts, achieving strong stabilization through cooperative effects, even when individual ligands exhibit low intrinsic binding affinity. Polyligand intercalation induces structural changes in G4 multimers, modifying their size and stiffness. Using a quantitative polymer theory framework, we connect these structural changes with the thermal stabilization of G4 multimers. Polyligands represent a general design principle for G4-targeting molecular stabilizers, offering enhanced G4 stabilization via programmable linker length and backbone rigidity.

## Graphical abstract

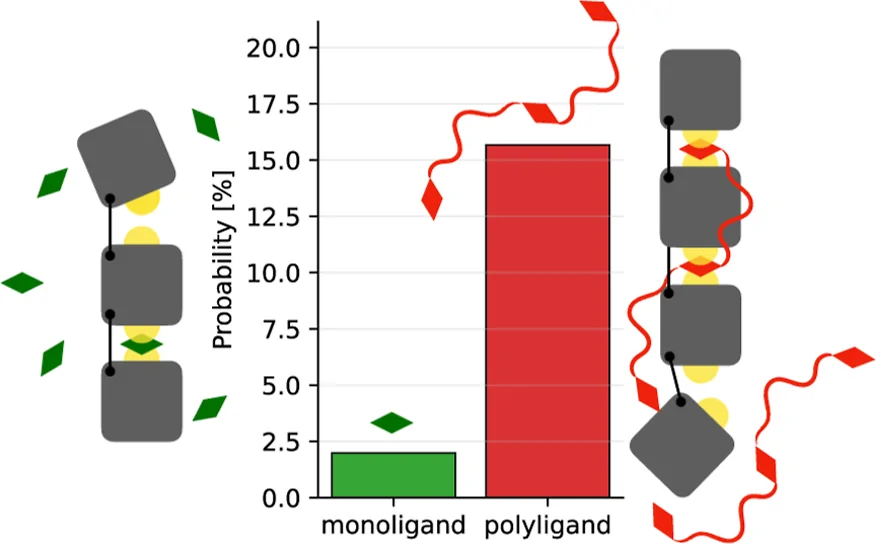

## Introduction

G-quadruplexes (G4s) are secondary structures formed by guanine-rich DNA and RNA sequences. From a structural point of view, a G4 consists of an array of quasi-planar G-tetrads stabilized by Hoogsteen-type hydrogen bonds, π-stacking interactions, and coordinating cations.^1–8^ Sequences capable of forming G4s are abundant in the genomes of higher eukaryotes^9–11^ and play a crucial role in regulating transcription, DNA replication, and cellular aging.^12–15^ In human telomeres, G-rich 3′-overhangs can extend from 50 to ≈600 nucleotides,^16,17^ and provide a scaffold for the formation of multiple sequential G4s, known as G4 multimers.^18^ This suggests G4 multimers with more than 20 G4s could possibly form. Indeed, in several papers, multimers long enough to form up to 12 G4s have been investigated both theoretically and experimentally.^19–22^ In particular, ref 19 showed *in vitro* that telomeric fragment sequences consisting of 48 TTAGGG repeats have a non-negligible probability to fold into multimers with 10 G4s. Stabilization of telomeric G4s via specific ligands has emerged as a promising therapeutic strategy to inhibit telomerase-mediated tumor cell proliferation.^23,24^

Despite extensive efforts to develop G4-stabilizing drugs experimentally^25–31^ and to characterize ligand-G4 binding motifs via atomistic simulations,^32–42^ achieving structural stabilization and selectivity remains challenging. Many of the G4-targeting ligands used in current *in vitro* studies exhibit comparable binding affinities toward G4 and duplex DNA motifs, due to their planar aromatic structures, leading to poor selectivity and potential off-target cellular toxicity.^43–48^ Moreover, while binding to individual G4s has been extensively characterized, much less is known about how ligands bind to G4 multimers and modulate their polymeric properties. Crucially, it is unclear how ligand-induced G4 multimer property changes relate to thermal stabilization. Recently, ligands have been shown to strongly affect the flexibility of G4 multimers *in silico.* ^49^ However, the connection between these polymeric effects and G4 thermal stabilization remains unclear.^20,49–51^ Progress in this direction is hampered by both experimental and computational limitations. Long G4-forming sequences are prohibitively difficult to study *in vitro*,^20^ while computational approaches capable of probing the equilibrium properties of long G4 multimers remain scarce.^52,53^ Addressing these challenges calls for a framework that treats G4 multimers as polymeric entities whose large-scale conformations and thermodynamics can be systematically analyzed.^49,54–56^

In this work, building on our recently developed tunable coarse-grained (CG) models for G4 mono- and multimers,^49,54^ we propose a novel class of ligands, which we call “polyligands”, defined by their multivalent, polymer-like architecture rather than by a specific chemical structure. Instead of relying on high ligand-G4 binding affinity, these polyligands are designed to achieve enhanced intercalation efficiency and, potentially, selectivity,^57^ through a multivalent approach, a widely recognized strategy to significantly increase effective affinity through multiple interactions.^58–60^ Using long-timescalescale coarse-grained molecular dynamics simulations, we investigate how polyligands with varying monomer number, backbone stiffness and ligand-G4 binding affinity interact with G4 multimers, revealing that architectural matching between ligand design and G4 periodicity enhances intercalation efficiency through cooperativity. We observe that ligand intercalation induces a structural transition toward elongated, rod-like G4 multimer conformations. We establish a theoretical framework, rooted in polymer theory, that quantitatively connects these ligand-induced structural changes to thermodynamic stabilization. Our results also suggest that polyligands could exhibit enhanced selectivity toward G4 multimeric architectures, exploiting the unique structural periodicity of G4 multimers by rational tuning of polyligand linker length and backbone rigidity.

## Results and Discussion

In our coarse-grained framework, a “polyligand” is a polymer-like macromolecule in which N monomeric G4-binding molecules (ligands) are covalently linked together by inert, flexible linkers. The specific chemical details are coarse-grained into effective interaction potentials (see Methods), to allow us to simulate system sizes and reach time scales necessary to capture the emergence of cooperative intercalation. Polyligands have two additional key control parameters compared to monoligands, namely, the stiffness of the polymeric backbone and the average intermonomer (ligand) spacing. A schematic representation of our G4 multimer and polyligand models is provided in Figure 1.

**Figure 1:**
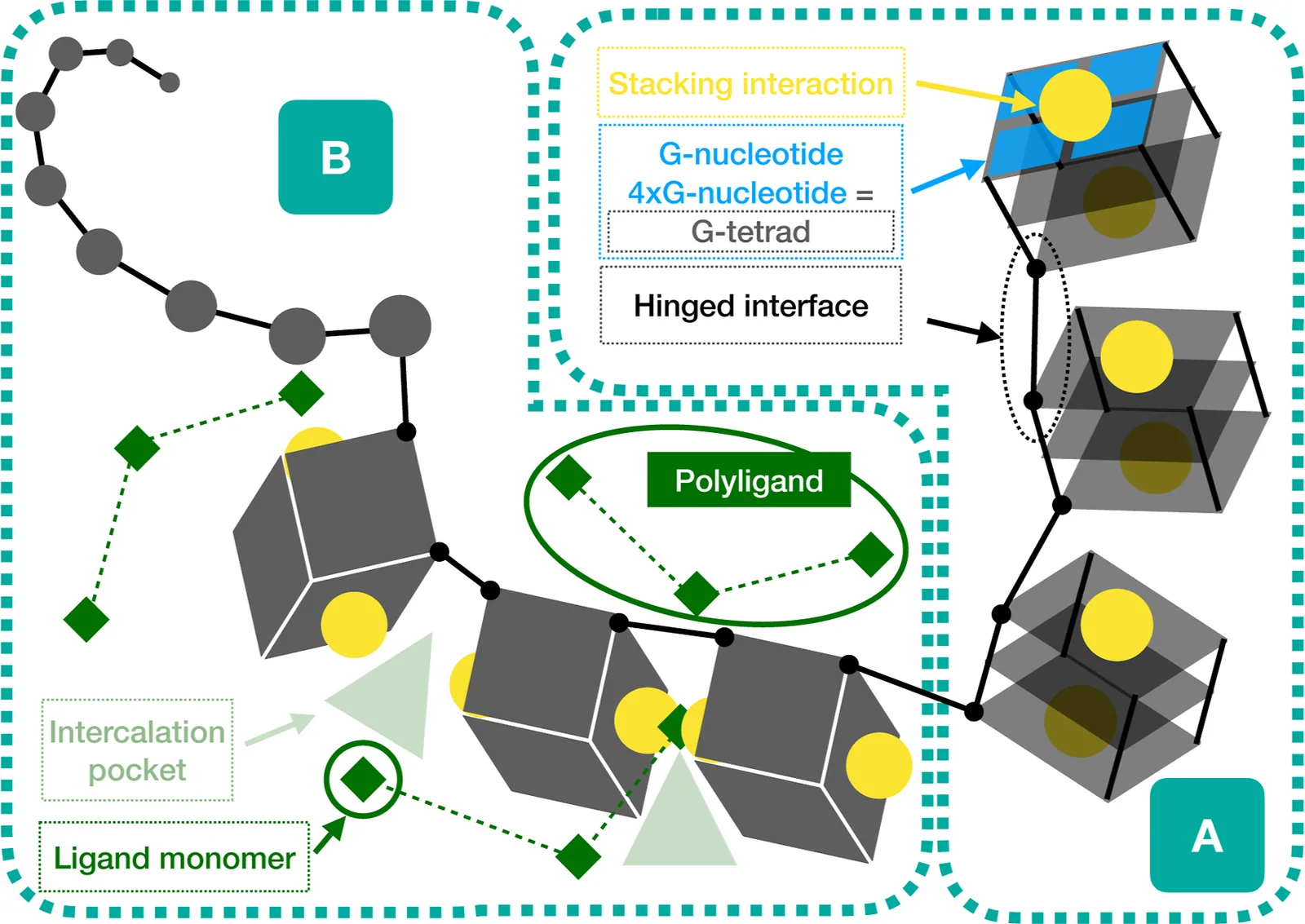
Schematic representation of polyligands intercalating into a G4 multimer. The representation of G4 monomers along the backbone is progressively simplified to reduce visual complexity. Panel A: a G4 multimer consisting of permanently bonded G4 monomers, joined by pairwise hinged interfaces. The central attraction between adjacent G4 monomers (i.e., stacking interaction) is represented by yellow spheres. Each G4 monomer is composed of three G-tetrads (gray planes). The links between them are represented by black lines. Four in-plane G-nucleotides (highlighted in blue) linked by Hoogsteen hydrogen bonds (not shown) constitute a G-tetrad. Panel B: The space between a pair of stacking sites at each hinged interface is defined as an intercalation pocket (light green triangles). Ligand monomers (green diamonds) are driven to the intercalation pockets via a central attraction. A ligand is intercalated if it is located between a pair of stacking sites (as shown in the lower right corner of panel B). Linking (depicted via dashed green lines) ligand monomers into a polymer-like structure constitutes a polyligand (polyligands with 3 monomers shown).

In this study, we consider the intercalation of ligand monomers into the pockets situated between adjacent G4s as the primary and only binding mode. Therefore, we use the term “ligand-G4 binding affinity” interchangeably with “intercalation affinity” throughout this work. This process is driven by the interaction between the ligand and the stacking sites of the outer tetrads of the G4 monomers. Although ligand–G4 binding occurs via multiple modes, focusing on a specific binding mode allows us to disentangle polymeric cooperativity from chemical specificity and systematically investigate how intercalated polyligands act as a physical scaffold, reinforcing and stabilizing the flexible “beads-on-a-string” arrangement of the G4 multimer, while identifying design rules that are transferable across ligand classes. All effects reported here arise from cooperative intercalation along the G4 backbone and are therefore not tied to the specific multimer length used in the simulations. Here, we simulate G4 multimers with 15 G4 monomers as a proof-of-concept system. This monomer number was chosen as representative of a long G4 multimer that can still reasonably fold from telomeric DNA G4-forming sequences, while being long enough for polymeric property variation along the backbone to be scrutinized. In the present simulations, the 15-mer contains 14 intercalation pockets, which is approximately 3× larger than the number of ligand monomers in the longest polyligand considered here (*N* = 5). We therefore expect the cooperative intercalation and stabilization mechanisms discussed below to apply to G4 multimers of comparable or larger relative size. For shorter multimers, where the number of available intercalation pockets becomes comparable to the number of monomers in the polyligand, the polyligand advantage is expected to decrease because the number of possible cooperative intercalation events becomes limited.

### Cooperative Intercalation of Polyligands in G4 Multimers

The idea behind cooperativity is simple: once the first monomer in a polyligand intercalates, the polyligand becomes tethered to the G4 multimer. Consequently, the remaining monomers in the same polyligand explore a significantly restricted phase space. If the polyligand backbone is tuned to match the spacing between the intercalation pockets, the unbound monomers in the now tethered polyligand will be positioned in the vicinity of intercalation sites, thereby increasing the likelihood of successive intercalation events.

Figure 2 shows intercalation efficiency, defined as the fraction of ligands intercalated into a G4 multimer, for different G4-ligand affinities ϵ and polyligand monomer number *N*, as a function of simulation time. It can be observed that intercalation efficiency depends on both ϵ and *N*. For ϵ = 5, the ligand-G4 binding affinity is too low and ligands do not intercalate. At ϵ = 10, while monoligands (*N* = 1) intercalate with a low probability, polyligand variants *N* ∈ (3, 5) exhibit a dramatic increase in intercalation efficiency (approximately 20 and 30% for *N* = 3 and 5, respectively). This enhancement reflects the aforementioned cooperative mechanism rooted in the multivalency of the polyligand. The signature of cooperativity lies in the higher probability of finding polyligands with a larger number of intercalated ligand monomers, as shown in Figure S1. The primary penalty to translational entropy arises from the tethering of a polyligand to a G4 multimer. Successive intercalations of the remaining unbound ligand monomers result in additional losses of configurational entropy of the chain; however, since the phase space explored by the ligand monomers is concentrated in the vicinity of the intercalation sites, subsequent configurational entropy losses diminish and the overall binding free energy gets increasingly favorable as intercalation proceeds. Figure 3 shows snapshots of the simulations of G4 multimers with ligands, providing a visual representation of the enhancement in intercalation efficiency obtained with increasing polyligand length. In particular, Figure 3e shows an *N* = 5 polyligand, for ϵ = 10, progressing from a partially intercalated to a fully intercalated state within the same G4 multimer, illustrating the mechanism described above.

**Figure 2:**
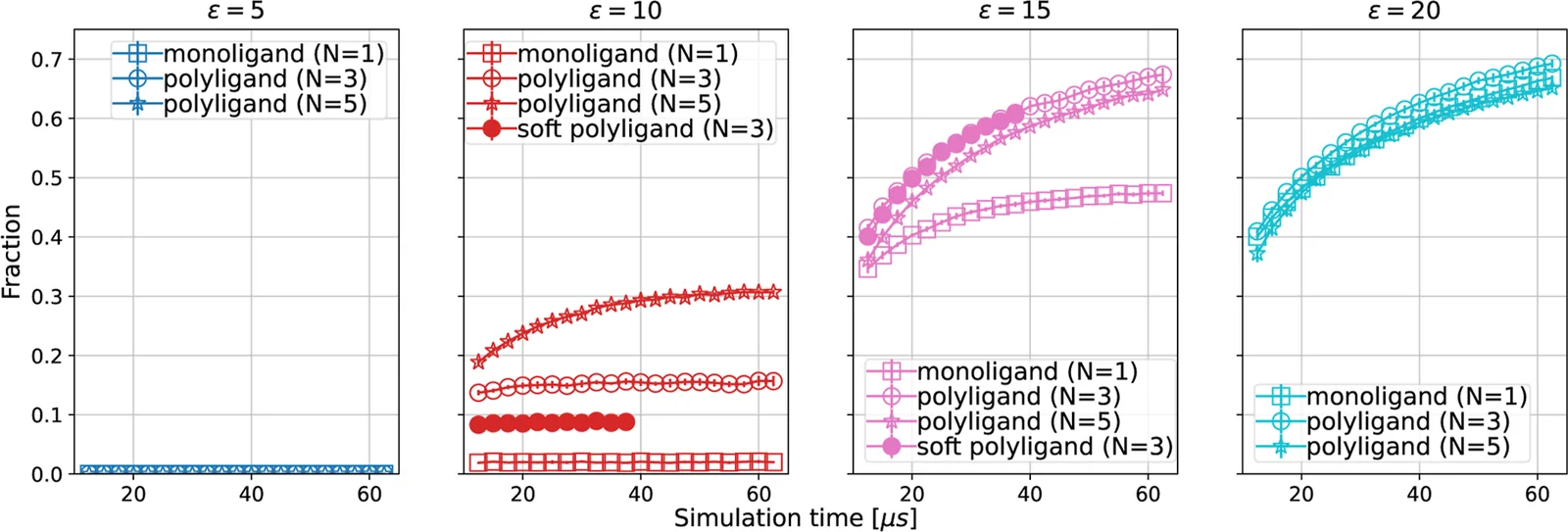
Fraction of ligands intercalated (relative to the total number of ligands in the simulation box), as a function of simulation time. A ligand monomer is considered intercalated if two and only two stacking sites (that constitute an intercalation pocket) can be found in its vicinity, within a cutoff radius of 1.3σ. Each panel features a comparison between different ligand variants, as annotated in the legend. Different panels correspond to a different intercalation affinity (from left to right ϵ ∈ {5, 10, 15, 20}). Error bars, calculated as the standard deviation of simulated data, are shown but are in places smaller than the symbol size.

**Figure 3:**
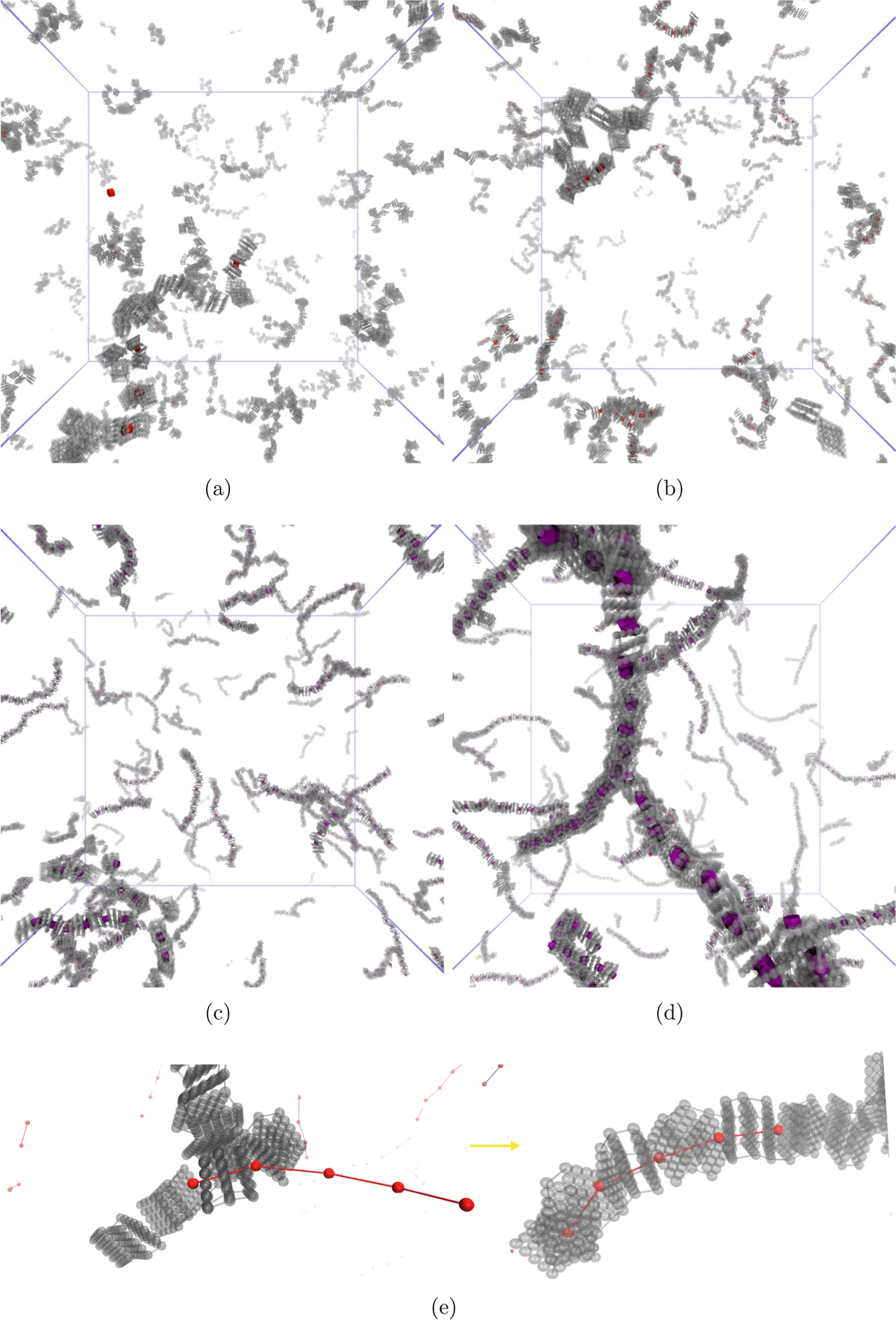
(a–d): representative simulation snapshots of G4 multimers interacting with monomeric and polymeric ligands. Intercalation events are highlighted with red cylinders for (a) ϵ = 10, *N* = 1; 3­(b) ϵ = 10, *N* = 3; and with purple cylinders for (c) ϵ = 15, *N* = 1; 3­(d) ϵ = 15, *N* = 5. 3 (e): representative intercalation event in which an *N* = 5 polyligand progresses from a partially intercalated to a fully intercalated state within a single G4 multimer, for ϵ = 10. Visualizations were rendered using the VMD molecular visualization program.^61^

With ligand-G4 binding affinity ϵ = 15, the overall intercalation probability increases further. In both *N* ∈ (3,5) cases, polyligands exhibit higher intercalation efficiency than monoligands due to cooperativity effects. However, we observe an inverse trend as a function of polyligand length, where the longer the polyligand, the lower the intercalation probability. Moreover, at ϵ = 20 the intercalation probability no longer increases when going from monoligands to polyligands. In both cases (ϵ = 15; ϵ = 20), polyligands are long and interactive enough for their monomers to intercalate into distinct G4 multimers simultaneously, leading to the bundling/branching observed in Figure 3c,d. The probability for bundling/branching as a function of ϵ is provided in Figure S2. The observed bundling/branching provides a possible explanation for the trends seen at both interaction strengths, as longer polyligands increasingly bridge neighboring G4 multimers, leading to multiple competing intercalation events and partially unbound ligand segments. Consequently, a fraction of the ligand monomers becomes unavailable for intercalation, reducing the overall intercalation efficiency. At the same time, stronger monomer-G4 interactions can compensate for the associated entropy loss of these bound states. Therefore, at ϵ = 20, the intercalation affinity of a monoligand can already exceed the entropy penalty, suppressing the cooperative multivalent effect. Note that intermultimer polyligand intercalation is highly unlikely to occur *in vitro* or in vivo, where concentrations are orders of magnitude lower than what we studied here.^17,44^ Polyligand intercalation should primarily occur through intramultimer interactions.

We proceed to consider whether and how the stiffness of a polyligand affects the intercalation efficiency. In order to do so, a soft *N* = 3 polyligand variant is considered as a targeted control for the role of backbone stiffness. These polyligands do not have backbone rigidity against bending and are hence soft compared to the standard polyligand variants studied here (see Simulation Methods for more detail). The informative comparison regimes are ϵ = 10, where cooperative intercalation occurs without pronounced bundling/branching, and ϵ = 15, where the system enters the bundling/branching regime. The simulations were stopped once the relevant intercalation behavior had been established: for ϵ = 10, the intercalated fraction had reached a stationary plateau; for ϵ = 15, the system showed the same qualitative time dependence as the corresponding standard polyligand in the bundling/branching regime. Figure 2 shows the corresponding intercalation efficiencies for the soft *N* = 3 variant Method, at ϵ = 10 and ϵ = 15. Notably, at lower intercalation affinity (ϵ = 10), softer polyligand variants exhibit a reduced intercalation efficiency compared with their more rigid counterparts. However, at higher intercalation affinity (ϵ = 15), the softness of the polyligand does not appear to play a role. This observation is in agreement with our previous reasoning: a more rigid backbone implies that the entropic loss associated with intercalation of consecutive monomers is lower, leading to a more pronounced cooperativity effect. Conversely, if the intercalation affinity of a monoligand is high enough to offset the entropy penalty, the benefit of cooperativity becomes secondary. These findings identify backbone stiffness as a crucial parameter in ligand design, in particular when the intrinsic ligand-G4 binding affinity is low to moderate.

Our results indicate that polyligands synthesized from monomers with a low/medium intrinsic binding affinity for double-helix DNA conformations (thereby minimizing potential side effects) could be a viable strategy for the development of selective G4-targeting ligands. While monomeric variants of such molecules would be ineffective for G4 stabilization, our results show that linking them into polymer-like macromolecules provides a significant enhancement in intercalation efficiency. This strategy introduces two new primary tuning parameters, namely the intermonomer distance (average distance between subsequent intercalation pockets) and backbone rigidity, which enable the rational control of intercalation affinity and selectivity toward specific G4 targets. In this framework, the gain in energy is driven by cooperativity, while selectivity is achieved by matching the intermonomer distance of a polyligand to the inter-G4 distance of a G4 multimer.

While our findings indicate that structural periodicity matching provides a mechanism to enhance selectivity, the present coarse-grained model does not resolve the chemical determinants required to discriminate between G4 multimers and duplex DNA. Additional simulations are required to fully characterize selectivity, where energetic differences between distinct G4 topologies, their conformational variability, and ligand-induced structural rearrangements, are resolved. Another consideration is that polyligands composed of more than a few monomeric ligands may be too large to efficiently access the cell, thereby limiting the enhancement in efficiency obtained through polymerization. To overcome this limitation, one could envisage the self-assembly of polymeric ligands from their constituent monomers inside the cell, where the formation of permanent bonds between monomeric ligands could be triggered. From a synthetic perspective, the polyligands described here should be viewed as short, monodisperse oligomers rather than as high-molecular-weight polymer drugs. The realistic synthetic feasibility of this concept is supported by the availability of G4-binding cores bearing peripheral amino, carboxylate, alkyne, or azide groups, which can, in principle, be connected through oligo­(ethylene glycol), alkyl, or peptide spacers using established conjugation strategies, including amine coupling and azide–alkyne cycloaddition. Such modularity would allow the interligand distance, linker composition, and local rigidity to be varied in a controlled manner, without requiring a long polydisperse polymer architecture. Several experimental precedents support this type of discrete multivalent design, including a tetrameric telomestatin derivative, dimeric metal–salphen complexes with polyether or peptide linkers, naphthalene-diimide dimers, and pyridostatin dimers tested against telomeric dimeric or multimeric G4 targets.^62–67^ Taken together, these studies indicate that the chemical space most closely related to the present coarse-grained model would consist of short oligomeric architectures, plausibly in the *N* = 2–5 range and therefore comparable in size to the *N* = 3 and *N* = 5 systems explored here. Such structures should be regarded as chemically plausible counterparts of the model, not as exact molecular reproductions of the coarse-grained representation. Nonetheless, they could capture the two design variables emerging from the simulations, namely interligand spacing and backbone stiffness, while limiting the size-related cellular-access issues expected for longer polyligands. For longer polyligand architectures, an alternative, more speculative route could be inspired by intracellular covalent assembly strategies, in which monomeric units are converted in situ into larger covalent oligomeric or polymeric structures.^68,69^

### Ligand Intercalation Modulates the Polymeric Properties of G4 Multimers

We now investigate how polyligand intercalation affects polymeric properties of G4 multimers. Figure 4 shows the normalized gyration radius *R*_g_^*^ = *R*_g_/*R*_g_^rod^ and the relative shape anisotropy parameter κ^2^ of the multimers as a function of polyligand lengths and ligand-G4 binding affinity. The (mass-independent) *R*_g_ is defined as

$$R_{g}=\sqrt{\lambda_{1}^{2}+\lambda_{2}^{2}+\lambda_{3}^{2}}$$

where λ_1_ > λ_2_ > λ_3_ are the eigenvalues of the gyration tensor

$$G_{\mu \nu }=\frac{1}{N}\sum_{i=1}^{N}\left(r_{i,\mu }-r_{cm,\mu }\right)\left(r_{i,\nu }-r_{cm,\nu }\right)$$

where *r*_i,μ_ and *r*_cm,μ_ are the μ-th Cartesian components of the position of the *i*-th particle and the center of mass, respectively, and *N* is the number of G4 monomers in a multimer. *R*_g_^rod^ is the *R*_g_ of the equivalent ideal rod conformation of the G4 multimer (fully straight with all interaction potentials at their respective interaction minima). On the other hand, the κ^2^ parameter is defined as

$$\kappa^{2}=\frac{1}{2}\frac{\lambda_{x}^{4}+\lambda_{y}^{4}+\lambda_{z}^{4}}{{\left(\lambda_{x}^{2}+\lambda_{y}^{2}+\lambda_{z}^{2}\right)}^{2}}-\frac{3}{2}$$

**Figure 4:**
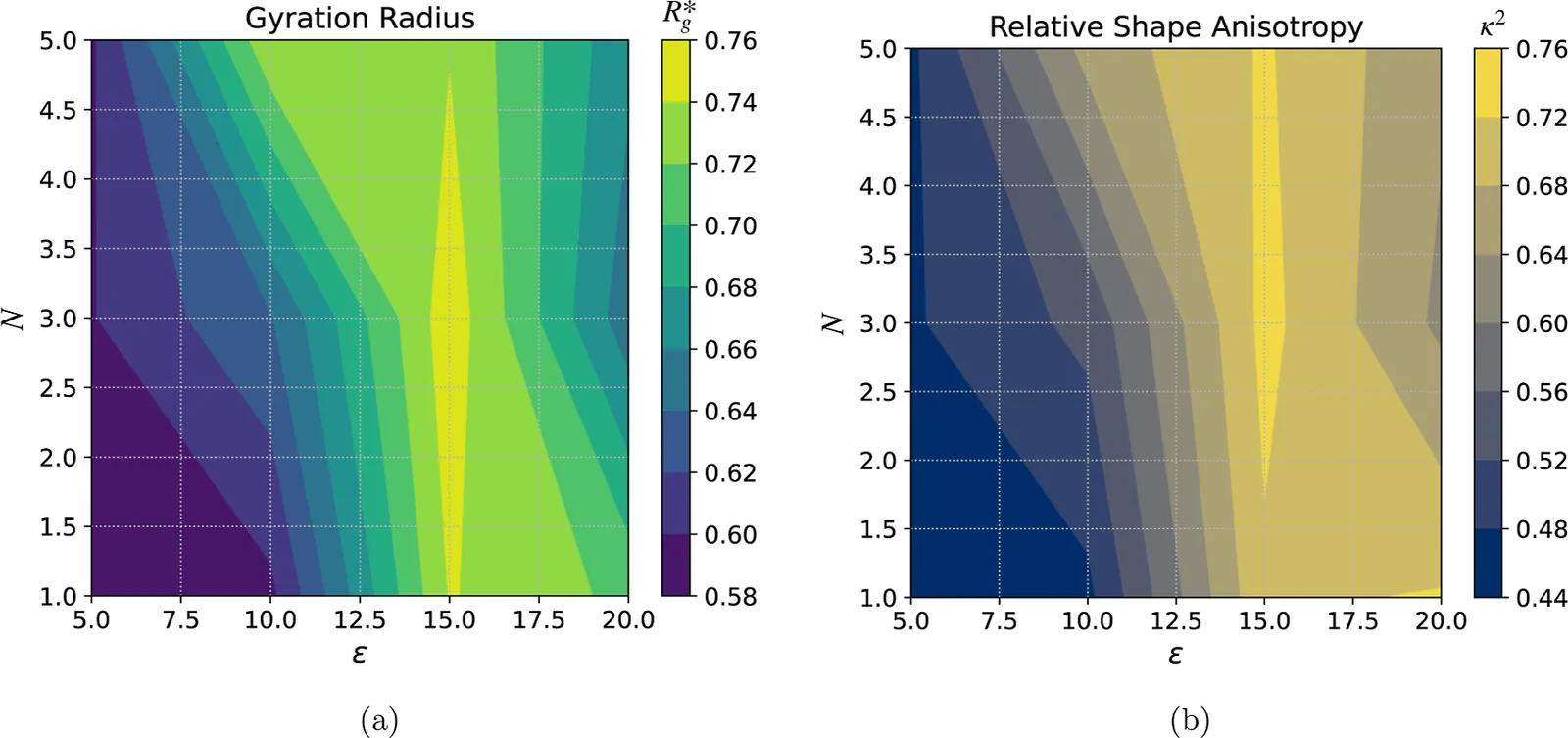
Contour plots of (a) the normalized gyration radius *R*_g_^*^ and (b) the relative shape anisotropy parameter κ^2^, as functions of polyligand monomer number *N* and intercalation affinity ϵ. In panel (a), *R*_g_^*^ is normalized by the gyration radius of the equivalent ideal rod-like G4 multimer conformation, with all bonds and interaction potentials at their respective minima. The relative shape anisotropy parameter is, by definition, normalized to unity.

As a descriptor of molecular symmetry, κ^2^ ranges from 0 (spherical distribution) to 1 (perfect linearity). It can be observed that both *R*_g_^*^ and κ^2^ exhibit a strong dependence on the number of monomers in the polyligand (*N*) and the ligand intercalation affinity (ϵ). While *R*_g_^*^ values vary by at most 30% (Figure 4a), κ^2^ displays a strong increase of up to 70% (Figure 4b). Both *R*_g_^*^ and κ^2^ reach a maximum at approximately ϵ = 15, followed by a decrease for higher ϵ values. This nonmonotonic dependence of both quantities on intercalation affinity is likely a consequence of the aforementioned bundling/branching, which becomes increasingly pronounced at larger ϵ. Focusing on ϵ = 10, i.e. the regime yielding optimal intercalation efficiency without branching, we observe that moving from *N* = 1 to *N* = 3 leads to an increase of *R*_g_^*^ of 10%, and a 27% increase in κ^2^, moving further to *N* = 5, *R*_g_^*^ increases by 28%, while κ^2^ increases by 54%. These findings indicate a ligand-induced transition of the G4 multimer from a coil-like to a rod-like configuration.

This transition reflects a change in the polymeric properties of the multimers. In the absence of ligands, G4 multimers behave like real polymers in a good solvent, adopting coiled, isotropic conformations to maximize entropy.^49^ As ligands intercalate, however, the attraction between ligand and G4 closes the pocket, effectively stiffening the backbone. Consequently, the *R*_g_^*^ does not change substantially, but the progressive occupancy of the intercalation pockets drives the G4 multimer toward an increasingly elongated rod-like conformation.

We further characterize the polymeric properties of G4 multimers undergoing intercalation by calculating the local persistence length *L*_p_ of G4 multimers. In real polymer theory, persistence length is a chain property that can vary substantially along the chain backbone. Schäfer and Elsner^70^ have shown that, to a very good approximation

1 $$\frac{L_{p}\left(k\right)}{L_{b}}=\langle \vec{a_{k}\;}\cdot \vec{R_{e}\;}/|\vec{a_{k}\;}{|}^{2}\rangle \approx \alpha {\left[\frac{k\left[\left(M-1\right)-k\right]}{M-1}\right]}^{2\nu -1}$$

where *M* is the number of G4 monomers in a multimer, $\vec{R_{e}\;}$ is the end-to-end distance vector, ${\mathrm{a⃗}}_{k}$ are vectors connecting each pair *k* of neighboring monomers along the backbone (i.e., bond vectors), *L*_b_ is the average bond vector length, α is a constant and ν is the exponent characterizing the linear dimensions of a G4 multimer in the solvent.^55,56^

Consistent with the observed transition to a rod-like morphology, an increase in intercalation efficiency is expected to correlate with enhanced structural stiffness. This is confirmed by the results of Figure 5 showing how the persistence length changes upon ligand intercalation. Overall, the trends mirror those observed for the intercalation efficiency in Figure 2. For ϵ = 10, polyligand variants more than double the persistence length of the G4 multimers. Interestingly, this trend inverts when the intercalation affinity is large enough to lead to bundling/branching, which likely disrupts the intrinsic chain stiffness. For this reason, the soft-polyligand persistence-length comparison is shown only for ϵ = 10, where cooperative intercalation occurs without pronounced bundling/branching and *L*_p_ can still be interpreted as a single-multimer stiffness measure. At moderate intercalation affinities, softer polyligand variants yield more flexible G4 multimers, in agreement with our earlier conclusion that backbone rigidity is a key determinant of cooperative intercalation. This observation highlights a complex interplay between the polymeric properties of the ligand and its intercalation efficiency; indeed, the global stiffening of the G4 multimer likely arises from a combination of the difference in backbone rigidity of the polyligand and the degree of occupancy of the intercalation pockets. The data clearly indicate that multimer stiffening is primarily driven by the fraction of intercalated ligands. Ultimately, these results suggest that polyligands act as molecular scaffolds, where the synergy between backbone rigidity and intercalation efficiency dictates the overall structure of the G4 multimers.

**Figure 5:**
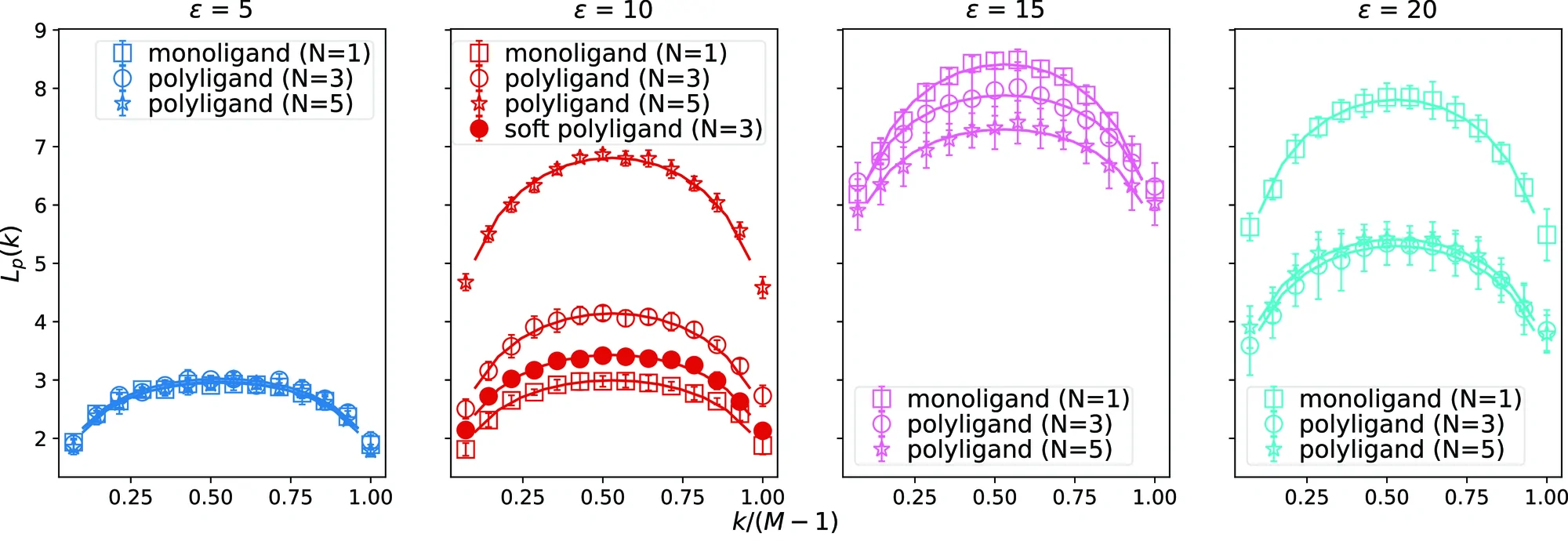
Local persistence length *L*_p_ (eq 1) of G4 multimers upon ligand binding, in units of average bond length *L*_b_ (calculated as the magnitude of vectors connecting the centers-of-mass of a pair of adjacent G4 monomers in a multimer). Symbol-ligand variant mapping is provided in the legend. Fits of eq 1 are shown as solid lines. The figure consists of four panels, each showing data for a particular intercalation affinity (increasing from left to right ϵ ∈ {5, 10, 15, 20}). Error bars represent the standard deviation of simulated data.

### Polymeric Properties Determine the Thermal Stability of G4 Multimers

We now investigate the link between the polymeric properties of G4 multimers and their thermal stability. Here, rather than treating stabilization as an energetic binding problem, we recast it as a polymer thermodynamics problem. Specifically, the framework is designed to estimate changes in melting temperature from ligand-induced changes in the polymeric properties of G4 multimers. The coarse-grained model used in this work is not designed to capture folding and unfolding transitions and therefore cannot directly simulate thermal melting. Using the thermodynamic state diagram in Figure 6, we can define the ligand-induced stabilization of G4 multimers in terms of Δ*F*_LB_(*T*) = *F*_FL_(*T*) – *F*_F+L_(*T*), under the assumption that the ligand stabilizes G4 multimers, Δ*F*_LB_(*T*) < 0. To connect this thermodynamic stabilization to structural observables, we propose a theoretical framework to estimate the change in melting temperature (Δ*T*) of G4 multimers based on polymer theory. Within this framework, the energetic stability gained from ligand binding is implicitly captured by the resulting changes in multimer stiffness (Kuhn length) and extension (end-to-end distance). Effectively, these structural parameters serve as a proxy for the increased stacking stability due to the intercalation process.

**Figure 6:**
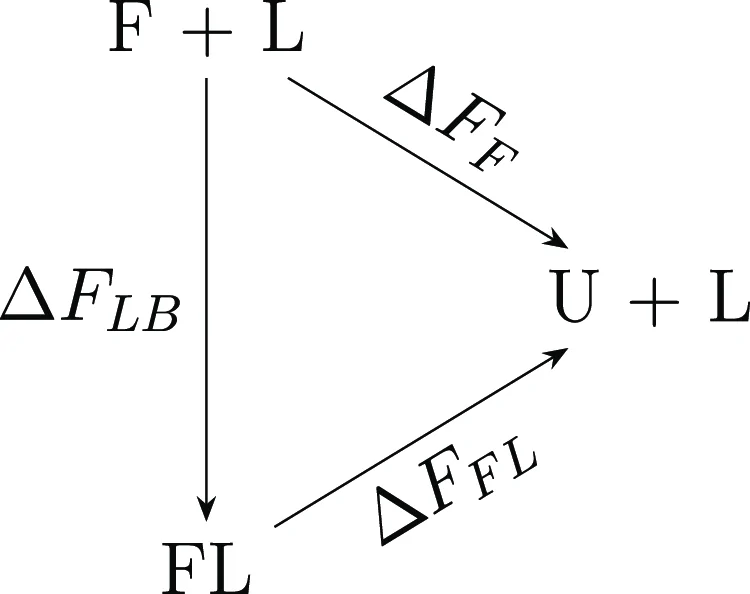
Thermodynamic state diagram illustrating the transitions between the different states of the G4 multimer and the ligand. The states shown are folded G4 multimer and ligand-bound (FL), unfolded G4 multimer + ligands (U + L), and folded G4 multimer + ligands (F + L). Δ*F*_F_ is the free energy change of unfolding the G4 multimer without bound ligands, Δ*F*_FL_ is the free energy change of unfolding the G4 multimer with bound ligands, and Δ*F*_LB_ is the free energy change of binding ligands to the folded G4 multimer. Each free energy change Δ*F* = Δ*U* – *T*Δ*S* is at the same temperature *T*. For the unfolding processes Δ*U*_F_, Δ*U*_FL_, Δ*S*_F_, Δ*S*_FL_ > 0. For the ligand-binding process Δ*U*_LB_, Δ*S*_LB_ < 0. F, U, and S are all state functions.

Considering that G4 multimers behave as real polymers,^49^ and that the action of ligands can be well represented as an effective increase in stacking interaction strength,^54^ it can be shown that the melting temperature change of G4 multimers under the action of ligands can be written in the following form

2 $$\Delta T=\frac{k_{B}T_{F}A}{\Delta S_{F}-k_{B}A}$$

where using Flory real polymer theory,^55^ *A* is

3 $$A=\frac{R_{F}^{2}}{Ma_{F}^{2}}-\frac{R_{FL}^{2}}{Ma_{FL}^{2}}+vM^{2}\left(\frac{1}{R_{F}^{3}}-\frac{1}{R_{FL}^{3}}\right)$$

In these expressions, *v*, *M* are fixed parameters, while *R*_FL_ and *R*_F_ represent average end-to-end distances of the G4 multimers with and without ligands, respectively. The corresponding Kuhn lengths are defined as *a*_FL_ = *R*_FL_^2^/*L*_FL_^cont^ and *a*_F_ = *R*_F_^2^/*L*_F_^cont^. *L*_X_^cont^ with *X* ∈ {FL, F} is the contour length of the folded G4 multimers with and without ligands, respectively. *T*_F_ is the melting temperature of the ligand-free multimer, and Δ*S*_F_ is the entropy loss due to the folding of a single-stranded G4-forming DNA sequence into a G4 multimer. Notably, as this folding process involves a significant reduction in conformational degrees of freedom, it occurs at a high entropic cost (Δ*S*_F_ ≫ 0). A detailed derivation of eqs 2 and 3 is provided in Supporting Information.

We can make clear qualitative statements from eq 3. If the end-to-end distance increases as a consequence of ligand intercalation (*R*_FL_ > *R*_F_), assuming constant stiffness (*a*_f_ = *a*_FL_ = *a*), we can distinguish two terms.

- Elastic penalty (entropic spring) term: an increase in the end-to-end distance results in a negative contribution to *A* $\left(\frac{{R_{F}}^{2}-{R_{FL}}^{2}}{Ma^{2}}<0\right)$ , which acts as a destabilizing factor.
- Excluded volume relief term: stretching the multimer results in a positive contribution to *A* $\left(\frac{vM^{2}}{{R_{F}}^{3}}-\frac{vM^{2}}{{R_{FL}}^{3}}>0\right)$ , which exerts a stabilizing effect.

Thus, *A*, and hence Δ*T*, are determined by the competition between elastic penalty and excluded-volume relief. According to eq 2, for a ligand to increase the melting temperature of a multimer (Δ*T* > 0), the stabilization parameter *A* must be positive and the unfolded state should have a larger entropy than the ligand-stabilized folded state (i.e., Δ*S*_F_ – *k*_B_ *A* > 0). For *A* > 0 to occur, the increase in end-to-end distance (*R*_FL_ > *R*_F_) must be accompanied by a sufficient increase in Kuhn length (*a*_FL_ > *a*_F_). Physically, this implies that the potential energy gain from ligand intercalation must outweigh the entropic cost of increasing the end-to-end distance of the G4 multimer. Notably, this relation predicts that longer G4 multimers and those in better solvent will exhibit a larger A and hence a more pronounced thermal stabilization.

In Figure 7 we show how eq 3 behaves as a function of the end-to-end distance change *R*_FL_ – *R*_F_ and Kuhn length change *a*_FL_ – *a*_F_. The simulation data superimposed on the plot (black dots) confirm the trends discussed in the previous sections. For ϵ = 5, the intercalation affinity is insufficient to drive significant structural changes, providing no (relevant) increase in *A*. Conversely, for ϵ = 10, we observe a sharp increase in *A*, directly reflecting the boost in intercalation efficiency. This means that ligand intercalation leads to an increase in melting temperature. However, further increasing the affinity does not yield indefinite stabilization. For the *N* = 1 variant, across the various intercalation affinities investigated, we can see that *A* reaches a plateau and even slightly decreases between ϵ = 15 and ϵ = 20. For polyligands with *N* ∈ (3,5), this saturation is even more pronounced due to the onset of branching and bundling, as can be seen in Figure 3. Ultimately, our model demonstrates that thermal stabilization is intrinsically related to an increase in the end-to-end distance accompanied by an increase in G4 multimer stiffness. Ligand intercalation drives the transition toward an elongated, more rigid configuration, in which the binding energy compensates for the loss in conformational entropy. This framework provides a way to predict changes in melting temperature and therefore ligand-induced stabilization from experimentally accessible structural observables.

**Figure 7:**
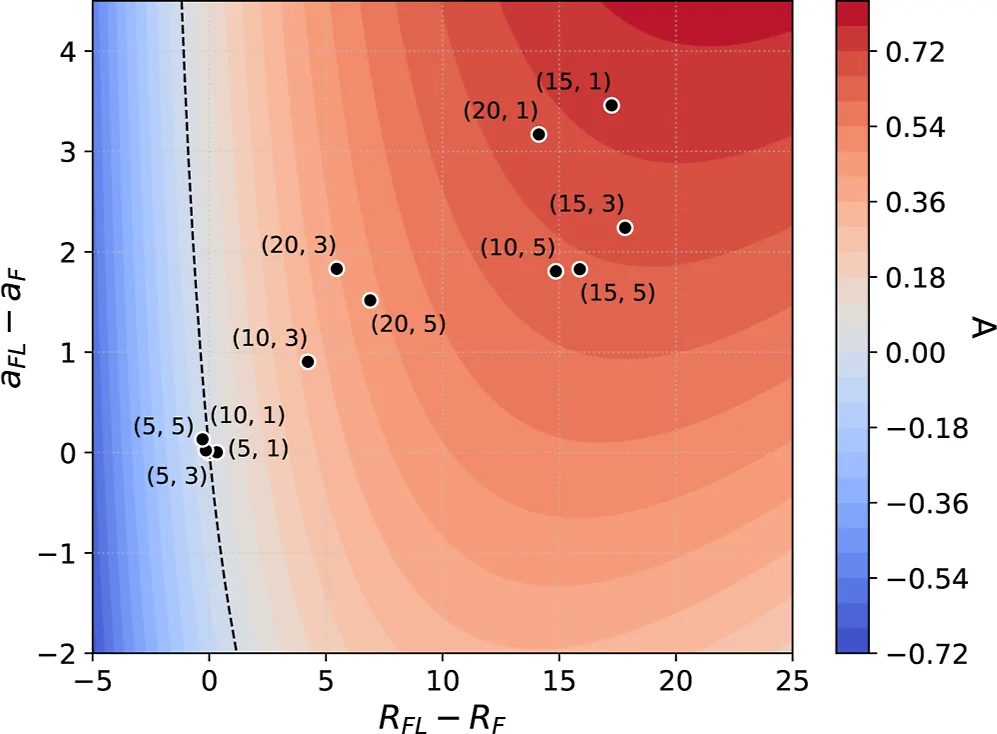
Contour plot of *A* (eq 3) as a function of the end-to-end distance difference between G4 multimers with and without ligands, *R*_FL_ – *R*_F_ (*x*-axis), and Kuhn length difference between G4 multimers with and without ligands, *a*_FL_ – *a*_F_ (*y*-axis). Kuhn length differences are given in units of average bond length *L*_b_. Otherwise, all values are provided in simulation units. Values that correspond to a particular (ϵ, *N*) parameter set from simulations are highlighted with black symbols, with the corresponding label in its vicinity (raw values are provided in Table S1). The solid black line denotes the contour for which *A* = 0. Color bar encodes the change in *A* (which can be scaled to melting temperature change by a factor *k*_B_ *T*/Δ*S*_F_).

## Conclusion

The central question addressed in this work is how efficiently polymeric variants of ligands stabilize long G4 multimers, in comparison to the corresponding monomeric ligand variant. Using long–timescale coarse-grained simulations, we quantified how polyligand architecture—monomer number (*N*), intercalation affinity (ϵ), and backbone stiffness—is related to intercalation efficiency and the emergent polymeric properties of G4 multimers. We further connected these polymer-level changes to thermal stabilization through a simple thermodynamic framework.

At low-to-moderate (ϵ = 10) intercalation affinity, polyligands intercalate more efficiently than monoligands, demonstrating a clear cooperativity-driven enhancement of intercalation (Figure 2). Intercalation closes the hinged interface intercalation pockets between adjacent G4s, driving a transition toward rod-like conformations (Figure 4). These conformational changes coincide with substantial backbone stiffening: polyligands can more than double the G4 multimer persistence length relative to the ligand-free case (Figure 5). For equal *N* and ϵ, stiffer polyligands intercalate more efficiently than the flexible ones, although this distinction disappears at higher ϵ, where monomer-level intercalation affinity dominates the behavior. For strong intercalation affinities (ϵ ≥ 15), the polymeric ligands can cause bundling/branching of G4 multimers, indicating a regime where strong local interactions override cooperative effects. We derived an explicit link between polymer properties and stabilization, defined here as the increase in melting temperature. Thermal stabilization of a G4 multimer is governed by a delicate balance: the increase in stiffness must compensate for the entropic penalty of intercalation-induced elongation. Equation 3 captures this balance, and demonstrates that at low-to-moderate intercalation affinity (ϵ = 10), polyligands yield a greater increase in melting temperature than their monomeric counterparts. Importantly, very high intercalation affinity does not necessarily enhance stabilization, and can even reduce the melting temperature, highlighting the nontrivial interplay between enthalpic gains and entropic costs. Taken together, these results demonstrate that the synthesis of short, relatively rigid polyligands—polymer-like ligand macromolecules—from ligands with low/modest ligand-G4 binding affinity is a promising strategy to enhance intercalation through cooperativity. Moreover, matching the average interligand spacing to the characteristic length scale of a G4 provides a potential route toward selectivity, bypassing the need for strong monomer–DNA interactions, which are often the primary drivers of off-target duplex binding. Because this mechanism relies only on periodic binding sites and polymer connectivity, it is expected to apply to other repetitive biomolecular targets beyond G4 DNA. Our findings emphasize that ligand efficacy can emerge from polymer-level cooperativity and architectural matching, rather than from strong local interactions alone, offering a powerful design strategy for next-generation G4 stabilizers and, more broadly, pointing toward a new design principle for the selective targeting of repetitive nucleic acid assemblies based on multivalent topological recognition. For example, polyligand-induced stabilization of G4 multimers could be a viable new strategy for anticancer therapeutic drug design, relevant for telomerase activity regulation in cancerous tissues.^29^ The same cooperative intercalation mechanism could be exploited to architecturally “rectify” beads-on-a-string G4 assemblies into nanowire-like scaffolds, suggesting potential applications in bioinspired nanostructured materials.^71–76^ Our framework is a scalable and transferable way to relate ligand architecture to G4 multimer stabilization. Additional environmental factors, such as molecular crowding—which has been shown to influence both the equilibrium conformations of G4 and ligand–G4 interactions^77^—can be incorporated within the present framework to explore cooperativity-enhanced stabilization under more complex and physiologically relevant conditions.

## Methods

### Modeling Details

We perform molecular dynamics simulations using our CG model of G4 multimers, originally presented in Mostarac et al.,^49^ where more details can be found. The baseline structure in our simulations is the G-tetrad, modeled as a 5 × 5 grid of equidistant spheres. The excluded volume of a sphere with a diameter σ is realized via the Weeks–Chandler–Andersen (WCA) potential^78^

$$U_{WCA}\left(r\right)=\{\begin{array}{ll} U_{LJ}\left(r\right)-U_{LJ}\left(r_{cut}\right), & \text{if}r<r_{cut}\\ 0 & otherwise \end{array}$$

where *U*_LJ_(*r*) is the conventional Lennard–Jones (LJ) potential

4 $$U_{LJ}\left(r\right)=4\epsilon \left\{{\left(\sigma /r\right)}^{12}-{\left(\sigma /r\right)}^{6}\right\}$$

where the cutoff value is *r*_cut_ = 2^1/6^σ. The parameter ϵ defines the interaction strength (relative to the thermal energy scale). Note that we report the ϵ and σ parameters with subscripts WCA or *LJ* to help distinguish parameter values used for the WCA and LJ interactions, respectively. A G4 monomer consists of three G-tetrads, linked together via finitely extensible, nonlinear elastic (FENE) bonds^79^

5 $$U_{FENE}\left(r\right)=-\frac{1}{2}K\Delta r_{\max}^{2}\;\ln\left[1-{\left(\frac{r-r_{0}}{\Delta r_{\max}}\right)}^{2}\right]$$

where *K* is the rigidity of the bond, Δ*r*_max_ is the maximal stretching length and *r*_0_ is the equilibrium bond length. Specifically, the corner particles in adjacent G-tetrads are linked. Making multimeric structures out of G4 monomers is achieved by introducing FENE linkers between a randomly chosen pair of corner particles on adjacent G-tetrads of neighboring G4 monomers. In order to mimic the stacking interactions between monomers, the center-of-mass (CoM) particles of the outer G-tetrads exhibit a central attraction, realized via the LJ interaction potential. These particles are referred to as stacking sites. The CoM particles within the same G4 do not have a central attraction between them. The LJ interaction (eq 4) is a good representation of the affinity monomers might have for the solvent and/or each other, and is often used in computational studies for this purpose.^80^ Moreover, it is a good choice to mimic the stacking interactions between G4 monomers and ligand-induced self-assembly of G4 monomers into multimers.^54^ Crucially, the effects of ligands on G4 multimers, as measured by scattering experiments, have been well captured by an effective central attraction potential.^81^ Tuning the stacking interaction is a simple but effective way to mimic the solvent in experiments, as long as one is exclusively interested in equilibrium properties.

Ligands are modeled as LJ spheres, attractive to the stacking sites in a G4 multimer and inert to all other particle types. The ligand shape/size does not af its intercalation probability in simulation (excluded volume associated with ligand monomers is negligible). This is of course a limitation, but one that we think mainly makes for quantitative differences. We do not expect nontrivial excluded volume associated with the shape of the ligand monomers to qualitatively impact intercalation, related properties, or the conclusions stated in this work. The reason for this is that ligands tend to be planar molecules with the longest dimension roughly similar to or smaller than the length of a G-quartet. As an example, one can compare the TMPyP4 porphyrin with a G-quadruplex monomer (see Protein Data Bank entry “2A5R”).^82^ Shape-related orientation constraints could have implications for how polyligands would need to be cross-linked to still allow for cooperative intercalation. Intercalation probabilities and related properties could be affected, when one considers the explicit chemical detail, including electrostatics. In this scenario, the intercalation binding mode would be in competition with other possible bonding modes. Having said that, as long as the ligands prefer the intercalation mode, we expect our conclusions to remain valid. In our simulations, intercalation is a reversible but not exclusive interaction, meaning that, while unlikely, it is in principle possible for two ligands to intercalate in the same stacking interaction pocket. Polyligands are realized by linking ligand particles via FENE bonds. Moreover, an isotropic bending pair potential *U*_bend_ between each subsequent three ligand units is used for the standard polyligands considered in this work. *U*_bend_ is given by

$$U_{bend}\left(\phi \right)=\frac{K_{b}}{2}{\left(\phi -\phi_{0}\right)}^{2}$$

where ϕ is the angle between the vectors spanning from particle *i* to its nearest neighbor particle pair (*i* – 1, *i* + 1), *i* ∈ [2, *N* – 1]. *K*_b_ is the bending constant, while ϕ_0_ is the equilibrium bond angle. We also considered soft polyligand variants, which, in comparison with the standard polyligand variants, do not have a bending potential to provide rigidity against bending. All polyligand variants we explored behave like real polymers in a good solvent, but the standard variants we explored have additional rigidity against bending (see details in Section on Units).

### Simulation Method

We performed MD simulations using the ESPResSo software package.^83^ The carrier fluid was represented implicitly, via the Langevin thermostat at fixed temperature *T*.^84^ In practice,this means that the Langevin equations of motion are integrated over time *t* numerically

6 $$M_{i}\frac{d{\mathrm{\nu ⃗}}_{i}}{dt}={\mathrm{F⃗}}_{i}-\Gamma_{Tl}{\mathrm{\nu ⃗}}_{i}+{\mathrm{\xi ⃗}}_{i}^{Tl}$$

7 $$I_{i}\frac{d{\mathrm{\omega ⃗}}_{i}}{dt}={\mathrm{\tau ⃗}}_{i}-\Gamma_{R}{\mathrm{\omega ⃗}}_{i}+{\mathrm{\xi ⃗}}_{i}^{R}$$

where for the *i*-th particle in eq 6, *M*_i_ is, in general, a rank-two mass tensor that, in our case, of isotropic monomers reduces to a scalar, ${\mathrm{F⃗}}_{i}$ is the force acting on the particle, ${\mathrm{\nu ⃗}}_{i}$ denotes the translational velocity. Γ_Tl_ denotes the translational friction tensor that once again in our particular case reduces to a single scalar friction coefficient. Finally, ${\mathrm{\xi ⃗}}_{i}^{Tl}$ is a stochastic force modeling the thermal fluctuations of the implicit solvent. Similarly, in eq 7, *I*_*i*_ denotes *i*-th particle inertia tensor (a scalar for a homogeneous sphere), ${\mathrm{\tau ⃗}}_{i}$ is the torque acting on it, ${\mathrm{\omega ⃗}}_{i}$ is the particle rotational velocity. As for the translation, Γ_R_ denotes the rotational friction tensor that reduces to a scalar, and ${\mathrm{\xi ⃗}}_{i}^{R}$ is a stochastic torque serving for the same purpose as ${\mathrm{\xi ⃗}}_{i}^{Tl}$ . Both stochastic terms satisfy the conditions on their time averages^85^

$${\left\langle {\mathrm{\xi ⃗}}^{Tl/R}\right\rangle }_{t}=0$$

$$\left\langle {\mathrm{\xi ⃗}}_{l}^{Tl/R}\left(t\right){\mathrm{\xi ⃗}}_{k}^{Tl/R}\left(t^{\prime }\right)\right\rangle =2\Gamma_{Tl/R}k_{B}T\delta_{l,k}\delta \left(t-t^{\prime }\right)$$

where *k*, *l* = *x*, *y*, *z*.

Forces and torques in eqs 6 and 7 are calculated from interparticle interaction potentials. Each simulation box contained 6120 G-tetrads, which were combined into 136 G4 multimers with 15 G4 monomers. This monomer number was chosen as representative of a long G4 multimer that can still reasonably fold from telomeric DNA G4-forming sequences (based on the reported range of nucleotide number in such sequences), which is long enough for polymeric property variation along the backbone to be scrutinized. Simulations were performed at a fixed G4 monomer concentration of *C* = 0.6 mM. In addition, we added 4080 ligand monomers, which is exactly 2× the number of ligands necessary to populate all available intercalation pockets in a simulation box. Depending on the monomer number in a ligand variant, *N* ∈ {1, 3, 5}, the number of ligand molecules in the simulation box can be obtained as 4080/*N*. We used periodic boundary conditions and a cubic simulation box to approximate infinite systems and extract bulk properties at equilibrium. For the integration, the velocity Verlet algorithm was used,^86^ with a time step of 0.01 (see for more detail section on the simulation units). In all cases, the initial configurations were generated so that both the positions and orientations of the largest predefined structures were appropriately randomized. We ensured that the system relaxes into an equilibrium configuration by running an integration cycle for 2 × 10^6^ integration steps. To obtain statistically significant results, we present averages over 20 uncorrelated data sets (last simulation snapshot, across 20 separate simulation runs).

### Reduced Units and Mapping to Physical Parameters

In this subsection, we give an overview of the units used in our simulations. The parameters used for the G4 multimer model were first published and justified in Mostarac et al.^49^ This single set of parameters was determined to match the experimental scattering intensities for the various telomeric sequences reported in Monsen et al.^50^ The time scale and length scale in our MD simulations are set to [*t*] = 1 × 10^–9^ s and [*x*] = 0.4 nm, respectively. Note that the length scale corresponds to σ_WCA_ = 1, which is the diameter of a single particle in the 5 × 5 grid of particles outlining a G-tetrad, in reduced units. The energy scale in the simulations is set to room temperature, *T* = 298.15 K, which corresponds to the Langevin thermostat reduced temperature of *k*_B_ *T* = 1 and steric repulsion strength ϵ_WCA_ = 1. The above-stated parameter choices uniquely define a mass scale, the value of which can is irrelevant and can therefore be considered arbitrary due to the scope of this work.

The central attraction strength between the stacking sites was set to ϵ_LJ_ = 5. The equilibrium distance between the stacking sites is set to σ_LJ_ = 2. Stacking sites do not have an excluded volume interaction associated with them otherwise. The equilibrium distance between ligand monomers and stacking sites is set to σ_LJ_ = 1. The ϵ_LJ_ between ligand monomers and stacking sites is the intercalation affinity variable that we called ϵ throughout the paper. Ligand monomers have a steric interaction with all other particles in the simulation with the parameters (σ_WCA_ = 0.5, ϵ_WCA_ = 1). Note that with these parameters, the ligand monomers have a negligible excluded volume associated with them. They cannot pass through other particles in the system, and they minimize their central attraction interaction energy with the stacking sites, when they are exactly in between a pair of stacking sites that are themselves at a distance that minimizes their central attraction interaction energy.

The factor *K* of the potential in eq 5 is set to *K* = 10 in reduced units, for all linkers (for both G4 multimers and polyligands). The equilibrium distance for all FENE linkers in a G4 multimer is *r*_0_ = 2, and their maximum extension is Δ*r*_max_ = 1.5*r*_0_. Note that this corresponds to the distance at which the stacking sites minimize their central attraction interaction energy. With these parameters, the aspect ratio of a CG G4 monomer in our simulations (the ratio of the longest, i.e. principal, to the shortest component of the gyration tensor, given in eq) aligns with the experimental aspect ratio of a G4 monomer folded from a Tel22 sequence, as reported in Libera et al.^87^ The experimental aspect ratio was determined by fitting the form factor of the monomer to that of a cylinder (the ratio of the cylinder height to the diameter of its base). The equilibrium distance for FENE linkers in a polyligand is *r*_0_ = 6, and their maximum extension is Δ*r*_max_ = 1.5*r*_0_. The factor *K*_b_ in bending potential equation is set to *K*_b_ = 10, and the equilibrium bond angle ϕ_0_ was set to ϕ_0_ = π. This equilibrium distance for FENE linkers in a polyligand was chosen so that if a single ligand in a polyligand is intercalated in the intercalation pocket of a G4 multimer, subsequent ligand monomers in that polyligand will explore the phase space in the vicinity of a neighboring intercalation pocket. In this way, one effectively incorporates a characteristic length scale of a G4 multimer in the structural properties of the polyligand.

## Supplementary Material



## Data Availability

Simulation data and processed analysis outputs underlying the analyses and figures are publicly available through Zenodo at DOI: 10.5281/zenodo.21905850. The simulation script, analysis notebook, and versioning information are available in the accompanying GitHub repository at https://github.com/stekajack/polyligand_data_JCIM. The simulation protocol, model parameters, computational environment, and external software dependencies are described in the manuscript and the repository documentation.

## Notes

Polyligand occupancy distributions characterizing cooperative intercalation; intermultimer bridging probabilities characterizing bundling/branching; tabulated data containing end-to-end distances, Kuhn lengths, and stabilization parameter *A* values for all combinations of intercalation affinity and ligand monomer number; derivation of the relation between G4 multimer polymeric properties and ligand-induced changes in melting temperature (PDF)
