# Supplementary material for: Rational Polyligand Design for G‑Quadruplex Multimer Stabilization
Source: J Chem Inf Model. 2026 Aug 29;66(17):11246–58. doi: 10.1021/acs.jcim.6c01189 (PMC13580136; doi:10.1021/acs.jcim.6c01189)
Supplement: Supplementary file 1 [file ci6c01189_si_001.pdf]

Supporting Information

Rational Polyligand Design for G-Quadruplex Multimer Stabilization

Deniz Mostarac,*,† Flavio Scipione,‡ Mattia Trapella,¶ Luca Bertini,¶ Valeria Libera,¶ Jussara Amato,§ Bruno Pagano,§ Lucia Comez,‖ Alessandro Paciaroni,¶ Laura Lupi,⊥ and Cristiano De Michele*,‡

†*School of Chemistry, University of Edingburgh, EH9 3FJ Edinburgh , United Kingdom*

‡*Department of Physics, University of Rome La Sapienza, 00185 Rome, Italy*

¶*Department of Physics and Geology, University of Perugia, 06123 Perugia, Italy*

§*Department of Pharmacy, University of Naples Federico II, 80131 Naples, Italy*

‖*CNR - Istituto Officina dei Materiali (IOM), 06123 Perugia, Italy*

⊥*Dipartimento di Matematica e Fisica, Università Roma Tre, 00185 Rome, Italy*

E-mail: deniz.mostarac@ed.ac.uk; cristiano.demichele@uniroma1.it

Characterizing cooperativity and bundling/branching

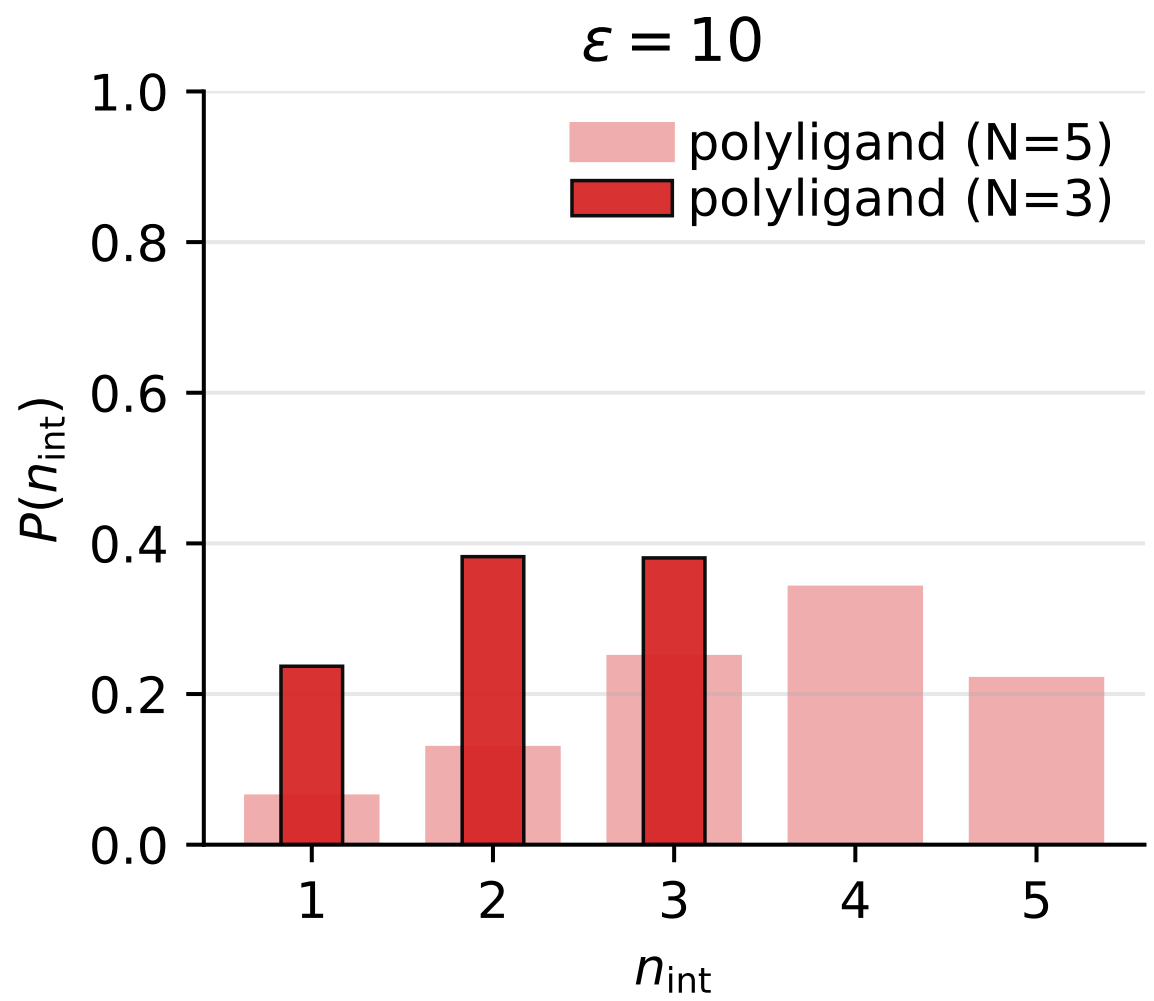

(a)

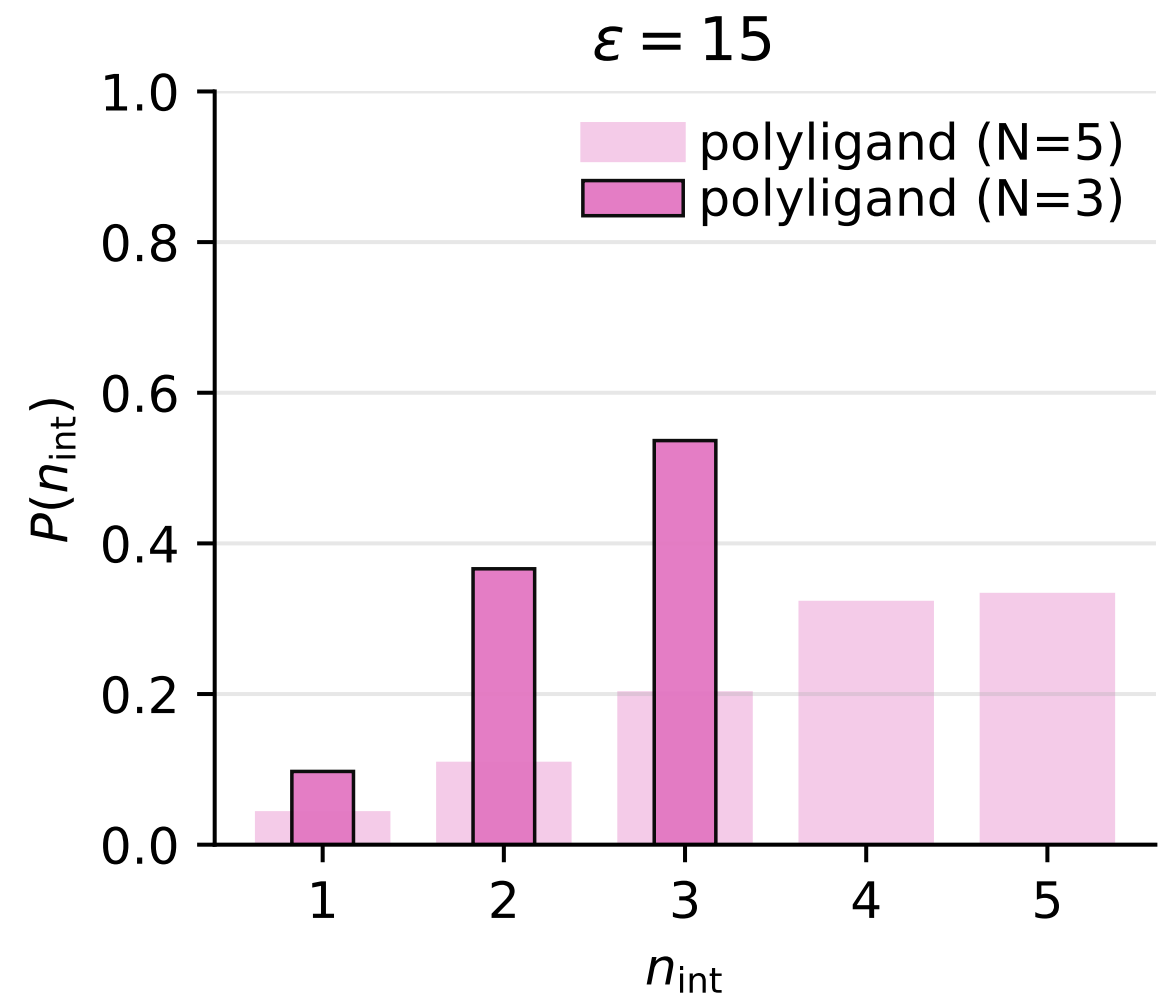

(b)

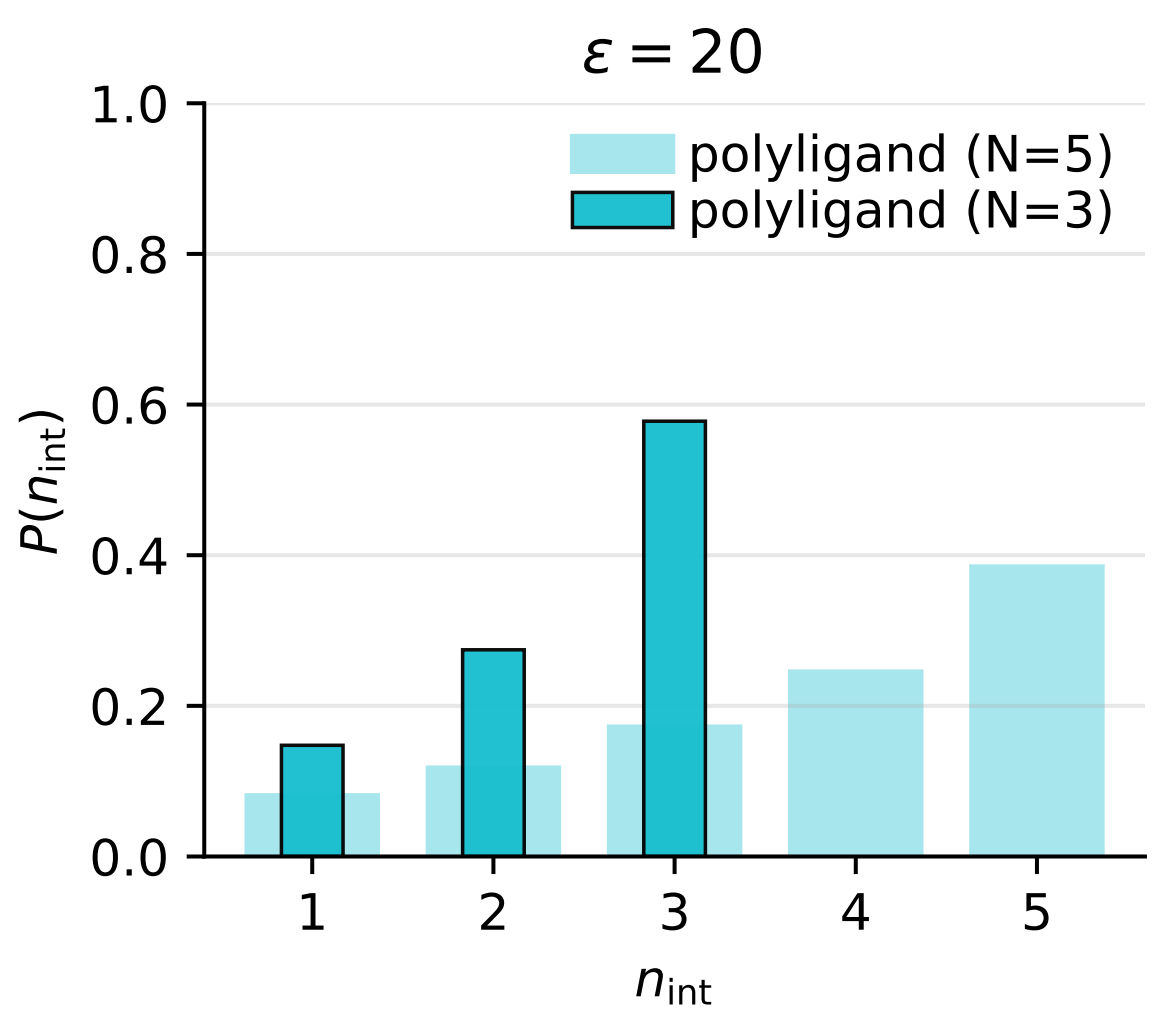

(c)

Figure S1: Bars show $P(n_{\text{int}})$, the probability that a polyligand has n_{int} intercalated monomers, for intercalation affinities (a) $\epsilon = 10$, (b) $\epsilon = 15$, and (c) $\epsilon = 20$. The unbound polyligand population is excluded before normalization. Polyligands with $N = 5$ are shown as wider transparent bars, while $N = 3$ polyligands are shown as narrower solid bars. The cooperative signature is the enhanced probability of finding polyligands with larger n_{int}.

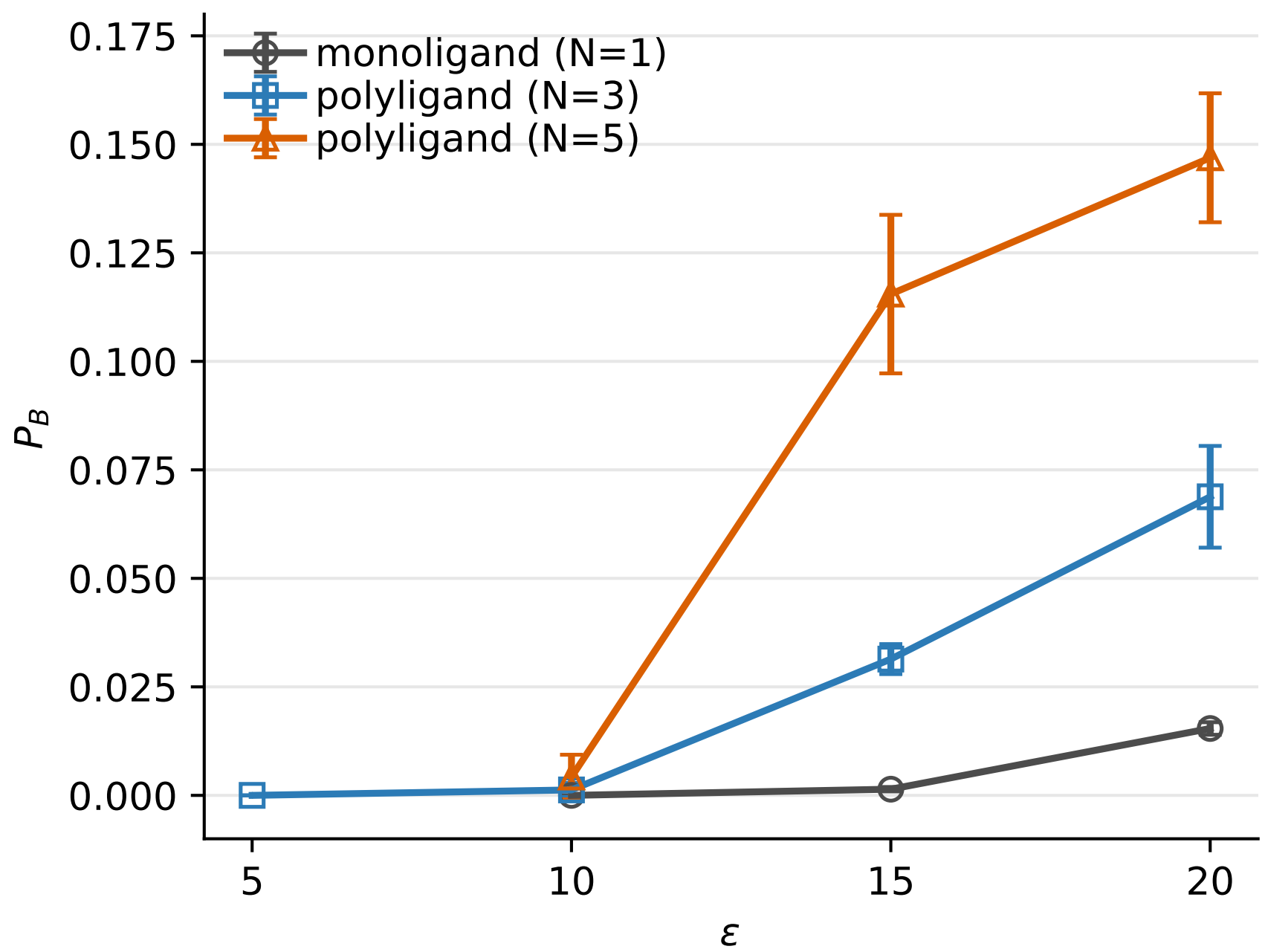

Figure S2: P_B denotes the probability that a ligand molecule intercalates simultaneously into more than one G4 multimer, leading to bundling/branching, as a function of intercalation affinity ϵ. Colour encodes the ligand variant, as indicated in the legend. The increase in P_B at high attraction strength, particularly for $N = 5$ polyligands, supports the formation of branched or bundled configurations.

Relating polymeric properties to melting temperature

Table S1: Table is showing (R, a, A) for each combination of ϵ and N. R and a calculated directly from simulated data, where $a =< R^2 > /L$ and $L = \sum_{k=1}^{N-1} < |a_k| >$. Excluded volume was estimated as the volume of an equivalent sphere how a diameter equal to the body diagonal of a G4 monomer, when all distances are such that the interaction potentials are at their respective minima.

$\epsilon \backslash M$	1.0	3.0	5.0
5.0	(37.98, 17.28, 0.00)	(38.31, 17.29, 0.03)	(37.84, 17.31, -0.01)
10.0	(37.69, 17.42, -0.02)	(42.21, 18.19, 0.32)	(52.84, 19.09, 0.64)
15.0	(55.23, 20.74, 0.74)	(55.81, 19.52, 0.68)	(53.86, 19.11, 0.65)
20.0	(52.11, 20.45, 0.70)	(43.45, 19.11, 0.42)	(44.88, 18.80, 0.46)

Derivation of the ligand-induced melting-temperature change

Lets write out the folding free energies (unfolded minus folded):

$$\Delta F_F(T) = F_{U+L}(T) - F_{F+L}(T),$$
$$\Delta F_{FL}(T) = F_{U+L}(T) - F_{FL}(T).$$

from which it follows that:

$$\Delta F_F(T) = \Delta F_{FL}(T) + \Delta F_{LB}(T). \tag{S1}$$

At some melting temperature T_F, the ligand-free folding free energy $\Delta F_F(T_F) = \Delta U_F - T_F \Delta S_F = 0$. Correspondingly, at some other melting temperature T_{FL}, the folding free energy with ligands present $\Delta F_{FL}(T_{FL}) = \Delta U_{FL} - T_{FL}\Delta S_{FL} = 0$. Taking that $T_{FL} = T_F + \Delta T$, where ΔT is small, we can expand both sides in Eq.S1 around T_F, and with a bit of algebra, reach an expression for the melting temperature increase due to ligands:

$$\Delta T = \frac{\Delta F_{LB}(T_F)}{\left(\frac{\partial \Delta F_F}{\partial T}\Big|_{T_F} - \frac{\partial \Delta F_{LB}}{\partial T}\Big|_{T_F} \right)}. \tag{S2}$$

Referring to the diagram of the relevant thermodynamic processes, shown in Fig. 6, it can be shown that Eq.S2 is equivalent to the following relation:

$$\Delta T = \frac{(\Delta U_{FL} - \Delta U_F) - T_F\left(\Delta S_{FL} - \Delta S_F\right)}{\Delta S_{FL}}.$$

Referring to the thermodynamic diagram, $\Delta U_{FL} - \Delta U_F = -\Delta U_{LB} > 0$, and this will be dominated by the energy change of interaction between the ligands and the molecule. Similarly, $\Delta S_{FL} - \Delta S_F = -\Delta S_{LB} > 0$, and this will include a contribution from the 'stiffness'

of the molecule. The equivalent relation $\Delta U_{LB} < T_F \Delta S_{LB}$ means that the loss of entropy on ligand binding must be compensated by the accompanying decrease in binding energy.

For demonstration purposes, let us first take a dummy "constrained" Flory free energy of a real polymer:

$$F(R,T) = k_B T \Phi(R); \quad \Phi = \frac{R^2}{M\sigma^2} - \frac{\pi \sigma^3 M^2}{6R^3} \tag{S3}$$

where we say that the Kuhn length $a = \sigma$ and is temperature independent (so M, σ are constants). At equilibrium for temperature T, we know that there is some equation $R^*(T)$ that solves:

$$\partial_R F(R,T)|_{R^*(T)} = 0 \tag{S4}$$

We can use this to see that:

$$\left.\frac{dF}{dT}\right|_{R^*(T)} = \frac{\partial F}{\partial R}\frac{\partial R}{\partial T} + \frac{\partial F}{\partial T} = \left.\frac{\partial F}{\partial T}\right|_{R^*(T)} \tag{S5}$$

Therefore, the implicit temperature dependence in the constrained free energy does not contribute to the first derivative of the minimized free energy. This is a template we can use to consider the general flory free energy expression, in which end-to-end distance and Kuhn length a are both T-dependent and mutually dependent:

$$F(R,a;T) = k_B T \Phi(R,a); \quad \Phi = \frac{R^2}{Ma^2} - \frac{\pi \sigma^3 M^2}{6R^3} \tag{S6}$$

M, σ are constants and a is related to R through some relation $a = \alpha(R)$. We can define the Lagrangian:

$$\mathcal{L}(R,a,\lambda;T) = k_B T \Phi(R,a) + \lambda[a - \alpha(R)]. \tag{S7}$$

At equilibrium (R^*, a^*, λ^*) for temperature T:

$$\partial_R \mathcal{L} = 0; \quad \partial_a \mathcal{L} = 0; \quad a = \alpha(R) \tag{S8}$$

Therefore, we can see that:

$$\frac{dF}{dT} = \frac{\partial \mathcal{L}}{\partial T}\bigg|_{R^*,a^*,\lambda^*;T} = k_B \Phi(R^*, a^*) \tag{S9}$$

We build on Eq. S2 by using the fact, that the folded states (with and without ligand) are defined as equilibrium minima of a Flory-type free energy of the form $F(R, a; T) = k_B T\, \Phi(R, a)$, where R is a size coordinate (e.g. end-to-end distance) and a is an effective stiffness parameter (e.g. Kuhn length), to arrive at the following:

$$\Delta T \;=\; \frac{\Delta F_{LB}(T_F)}{\Delta S_F - \frac{d\Delta F_{LB}}{dT}\big|_{T_F}} \;=\; \frac{k_B T_F\, A}{\Delta S_F - k_B A}, \tag{S10}$$

where A is:

$$A \;=\; \frac{R_F^2}{M a_F^2} - \frac{R_{FL}^2}{M a_{FL}^2} \;+\; vM^2 \left(\frac{1}{R_F^3} - \frac{1}{R_{FL}^3} \right), \tag{S11}$$

with v, M fixed, R_{FL} and R_F are end-to-end distances of the folded G4 multimers with and without ligands, respectively, and a_{FL} and a_F are Kuhn lengths of the folded G4 multimers with and without ligands, respectively. Note that the same algebra holds with Gibbs free energies at constant pressure ($U \to H;\ F \to G$).
